# Do clerkship schemes effectively improve pharmacy students’ understanding of and attitudes regarding pharmaceutical care?——a pre-post study in China Pharmaceutical University

**DOI:** 10.1186/s12909-019-1862-x

**Published:** 2019-11-15

**Authors:** Yuankai Huang, Dongning Yao, Weiwei Zhang, Yitao Wang, Wenbing Yao

**Affiliations:** 1 National Development Research Center of Licensed Pharmacist, China Pharmaceutical University, Nanjing City, Jiangsu Province, China; 2 State Key Laboratory of Quality Research in Chinese Medicine, Institute of Chinese Medical Sciences, University of Macau, Macau, China

**Keywords:** Pharmacy student, Clinical clerkship, Pharmaceutical care, Understanding, Attitudes

## Abstract

**Background:**

Clerkship is potentially an effective measure of improving the pharmacy students’ understanding of and attitudes toward pharmaceutical care. This research aimed to validate whether clerkship schemes in China effectively improve pharmacy students’ understanding of and attitudes toward PC, and discuss on how to improve the clerkship schemes for better quality.

**Methods:**

A pre-post and self-administrated questionnaire survey among three continuous years of students was conducted to measure Chinese fifth-year clinical pharmacy students’ differences in understanding of pharmaceutical care and attitudes toward pharmaceutical care before and after their clerkship schemes. Statistical analysis was conducted on the retrieved data.

**Results:**

602 respondents completed the post-part survey (drop rate = 1.8%). Correct rates of all statements regarding students’ understanding of pharmaceutical care were generally increased, but the rates of respondents holding correct understanding of the primary goal of pharmaceutical care (71.9 to 85.0%), the necessity of drug information support in pharmaceutical care (77.1 to 87.5%) and the patients’ role and duty when receiving pharmaceutical care (66.1 to 70.6%) were unsatisfactory before the clerkship and the improvement was not significant. Most statements regarding students’ attitudes toward pharmaceutical care were improved in general. However, rates of respondents holding negative attitudes toward students performing pharmaceutical care during their clerkship (3.7 to 7.5%) and choosing pharmaceutical care provider as their career after clerkship schemes (10.8 to 14.8%) had increased after the clerkship schemes.

**Conclusion:**

Clerkship generally improves clinical pharmacy students’ understanding of and attitudes toward pharmaceutical care, yet adjustments are also required in some contents of the clerkship for further improvements in its outcomes.

## Background

Pharmaceutical care (PC) providers’ understanding of and attitudes toward PC are two of the frequently studied [1–5] factors influencing the quality of PC practice [6–9]. PC providers’ understanding of PC is their perspectives of the definition, the primary goals, the value, the contents, the provision pattern and the required resources of PC, and their attitudes toward PC is the extent that they accept the desired and eventual outcomes of PC, as well as its processes [4, 5]. Improving the providers’ understanding of PC facilitates their comprehension of their roles and duties for achieving desired clinical outcomes, and improving the providers’ attitudes toward PC can generate internal incentives to enhance their professional capabilities and active engagement in PC, thus ultimately improve the quality of PC [10]. Evidence from several countries shows that clerkship schemes (clinical rotations, clinical practices), is an effective approach of improving providers’ understanding of and attitudes toward PC [11–15]. It is a practical course in which the to-be PC providers (i.e., pharmacy students) comprehensively observe and partially participate in the entire processes of hospital pharmacy practice, especially performing PC, during their higher education period, By undertaking a clerkship scheme, pharmacy students deeply participate in PC provision, through which their understanding of and attitudes toward PC can be improved [12, 16, 17].

The PC system in China is still in an early stage of development, and its quality is unsatisfactory in comparison to that in developed countries [18–25]. With various limitations, Chinese government is exploring approaches for improving the quality of PC [26–28]. Studies in China have indicated a certain extent of misunderstanding of PC by its providers, such as the concepts that taking the dispensing as the core of PC or the goal of PC is only to solve the drug-related problems raised by the clinicians, and reluctance of performing PC among some providers due to barriers like lacking support from hospital administrators or clinicians [29, 30], which may impair the quality practice of PC.

In China, most PC providers are clinical pharmacists who mainly graduated as pharmacy students from universities. Similar to medical students in China, pharmacy students are trained through classroom education, clerkship schemes and standardized hospital training [31, 32]. It should be noted here that the common definition of a pharmacy student in globe refers to students who are trained and professionally positioned as pharmacists or clinical pharmacists [33], but due to that in the past few decades the focus of pharmaceutical science in China was the research, development, manufacture and regulation of pharmaceutical products, while PC and the clinical pharmacy discipline were underemphasized until the most recent decade, the Chinese higher education system has incorporated higher education programs related to pharmaceutical research and development, production, circulation, supervision and healthcare provision into the scope of pharmaceutical education. Therefore, the meaning of “pharmacy student” is expanded to various types of pharmaceutical science students in the context of China [34]. To eliminate possible confusion generated from this difference and standardize the definition, this paper specifically uses the term “pharmacy student” solely for Chinese students majoring in clinical pharmacy in universities.

Clinical pharmacy programs of universities in China usually involve a clerkship scheme to reinforce pharmacy students’ professional knowledge and develop students’ clinical practice ability while deepening their understanding of PC and intensifying their recognition of the goals and value of PC [35–38]. The clerkships for pharmacy students are proved to be effective in improving the students’ cognitions and attitudes towards pharmaceutical care in several countries [12, 13]. However, the literature indicates that existing clerkship schemes in China are with potential defects in achieving the above goals, such as shortness of the clerkship period [39], disjunction and imbalance between classroom and practical training [40], and excessive clinical research tasks [41]. The absence of evidences on the effectiveness and defects of existing clerkship schemes in improving the students’ understanding of and attitudes toward PC is a possible reason for the hasty adjustments for the schemes without in-depth consideration regarding the circumstances of China by some clinical pharmacy programs.

This research aimed to examine the effectiveness of clerkship schemes in China on improving pharmacy students’ understanding of and attitudes toward PC, and discuss on how the clerkship schemes can be improved for better quality by design.

## Method

### Study design and participants

A cross-sectional pre-post and self-administrated questionnaire survey was conducted to collect data of the participants before and after their clerkship schemes.

The participants were fifth-year undergraduate students majoring in clinical pharmacy of three continuous years at China Pharmaceutical University (CPU), a pharmaceutical-science-focused university affiliated with the Chinese Ministry of Education. CPU is one of the top pharmaceutical universities and the first institution to offer a program in clinical pharmacy among China’s pharmaceutical higher educational programs [42]. It is a five-year program in which the last year is a clerkship period, during which the students are provided with basic training in clinical pharmacy practical and scientific research skills by teaching hospitals or facilities, and the arrangements and contents of this clerkship scheme are typical among clerkship schemes for pharmacy students in China [43]. Most clerkship schemes of other clinical pharmacy programs in China were mostly design based on it [35–38].

The inclusion criteria for respondents were as follows:

1. Registered as undergraduate fifth-year clinical pharmacy students in September 2014, September 2015 or September 2016 (All three groups shared almost the identical admission requirements, curriculum settings, teaching staff, clerkship arrangements and graduation requirements).
2. Had completed all the courses and passed all examinations or assessments before the clerkship (to eliminate the effect of quality issues related to the course education).
3. Had signed up for the clerkship schemes.
4. (For the post-part of the survey) had passed the final examination of the clerkship scheme.

Each respondent was assign with a distinct ID number to ensure the consistency of the pre-part and the post-part of the survey. Written consent forms were received from all the students who voluntarily participated in this study after they were informed about the aim and processes of the study orally and in writing and orally confirmed their willingness to participate.

### Instruments

A three-section questionnaire in Chinese was developed (Additional file 1).

1. Section 1 of the questionnaire concerns sociodemographic characteristics and records of study and training of clinical pharmacy, including the respondents’ gender, age, admission year, GPA, clerkship grades, grade of teaching hospital, and internship or work experience involving PC before the clerkship.
2. Section 2 contained 12 true-or-false questions about the definitions and goals of PC and the role and responsibility of pharmacists in providing PC, aiming to measure the correctness of the respondents’ understanding of PC. The questionnaire was designed according to the standardized definition and contents of PC [1], the guidelines regarding the standardized method for providing PC issued by American Society of Health-System Pharmacists and American College of Clinical Pharmacy [44, 45], and other studies concerning the understanding of PC [4, 5, 46]. Correct response to statement 3, 4, 10 and 12 was false.
3. Section 3 is 14-item 5-point Likert scale from 1 (strongly agree) to 5 (strongly disagree) concerning the respondents’ degree of acceptance of the goals, standard contents, modes and outcomes of PC and their PC workloads, targeting at the attitudes of pharmacy students toward PC and was built based on the Pharmaceutical Care Attitude Survey, which is a validated instrument that measures pharmacy students’ attitude toward PC [47], and was adapted in reference to other studies adopting or adapting this instrument [1, 4, 5, 9, 11, 14, 15] and context of China. Higher response scores indicate more positive attitudes, and statements 6 and 13 were reverse coded.

Two English-proficient native Chinese speakers and two native English speakers fluent in Chinese were recruited to translate the English reference. Then, under their assist, the draft questionnaire was reviewed and modified by experienced clinical pharmacists serving in hospitals for descriptive accuracy and readability in the social and linguistic context of China. In August 2014, 50 pharmacy students who had graduated in June 2014 were recruited for the pilot survey by e-mail. Researchers collected comments and suggestions regarding the readability and understandability of the questionnaire provided by 10 of those students and, again, modified the questionnaire. Then, the pilot survey data were collected from the other 40 students using the modified questionnaire. With the data above, the reliability of section 2 and 3 was separately assessed with Cronbach’s alpha, and the validity was assessed with the Kaiser-Meyer-Olkin measurement of sampling adequacy and Barlett’s Test of Sphericity The test results showed acceptable reliability (standardized Cronbach’s alpha: section 2 = 0.955, section 3 = 0.948) and validity (KMO measurement of sampling adequacy: section 2 = 0.595, section 3 = 0.609; Barlett’s Test of Sphericity: *p* = 0.000 for section 2, *p*_section3_ = 0.000 for section 3) of both instruments used in this study.

### Data collection

In August 2014, 10 first-year PhD students at CPU with clinical pharmacy backgrounds were recruited as the assistants of the researchers in data collection.

Data were collected through two rounds of self-administrated survey using paper questionnaires. Each respondent was required to complete the first round of the survey within one week before the clerkship and the second round of the survey within one week of after the clerkship by convenience of their time. To avoid interference from environmental factors, the students were required to finish the questionnaires in an undisturbed and quiet room provided by the researchers. The study was single blinded that the participants’ identification information was collected previous to the survey and kept by one researcher that didn’t contribute in data collection, and the assistants and the researchers contributed in data collection were blinded. Incomplete data were excluded.

### Data analysis

Descriptive statistics were used to display and analyze differences in the pharmacy students’ understanding of and attitudes toward PC before and after clerkship.

Kruskal-Wallis one-way analysis of variance test and the Mann-Whitney U test were used to examine whether there were significant differences in the pre-clerkship and post-clerkship understanding of and attitudes toward PC of respondents who differed by gender, registration year, clerkship hospital level (under current hierarchical health system of China, hospitals are divided into three levels, and hospitals with different levels have distinct functions and roles in the health system [48], and this may lead to differences in clerkship schemes for pharmacy students), internship or work experience involving PC, age, mean GPA before clerkship and mean clerkship grades.

Wilcoxon Signed Rank test were applied to examine the significance of the data regarding the differences in students’ understanding of PC before and after clerkship. McNemar’s test was used to examine the significance of the difference in the data between the students’ attitudes toward PC before and after clerkship. The difference was considered significant when the *p*-value was < 0.05.

All survey data were back-to-back cross-input into the computer by two of the authors using EpiData3.1 and analyzed using SPSS 24 (IBM Corporation, Armonk, NY, USA).

## Results

### Sociodemographic information

602 valid questionnaires were collected. 11 students were dropped because of failure to participate in or complete the clerkship, refusal to participate in our survey or other reasons (total response rate = 98.2%).

According to Table 1, 65.6% of the participants were female, and the average age was 24. The sample was evenly distributed among the three registration groups (32.9% in the 2014 registration group, 33.7% in the 2015 registration group and 33.4% in the 2016 registration group). The average GPA before the clerkship was dispersedly distributed around 3.1, with a standardized deviation of 1.8. The average clerkship grade was 84.7. Most students (81.6%) attended clerkships in tertiary hospitals, and only a small fraction of the respondents (7.0%) had internship or work experience involving PC prior to the clerkship.

**Table 1:** Respondents’ sociodemographic information

Item	Frequency (%) /mean (SD)
Gender	
Male	207 (34.4)
Female	395 (65.6)
Group by registration year	
2014	198 (32.9)
2015	203 (33.7)
2016	201 (33.4)
Clerkship hospital level	
Secondary hospital	111 (18.4)
Tertiary hospital	491 (81.6)
Internship or work experience involving PC	
Yes	42 (7.0)
No	560 (93.0)
Age	23.8 (1.3)
GPA before clerkship	3.1 (1.8)
Clerkship grade	84.7 (8.3)

Tests showed that students with different genders, registration years, clerkship hospital levels, ages, mean GPAs before clerkship and mean clerkship grades presented no significant differences in the understanding of and attitude toward PC either before or after the clerkship. However, a significant difference was found between the respondents with and without internship experience involving PC both before and after the clerkship.

### Respondents’ understanding of PC

As shown in Table 2, the overall correct rates increased during the clerkship. Statements 1 and 6 examined the respondents’ understanding of the roles and responsibilities of the pharmacist in PC provision; the correct rates for both were high (80.1 and 96.9%) before the clerkship and increased slightly thereafter. Statements 2, 4, 5 and 11 focused on the definitions and concepts of PC, and the correct rates were improved (71.9 to 85.0%, 46.0 to 68.6%, 68.1 to 88.7% and 86.4 95.9%), among which the correct rates for statements 4 (*p* = .02) and statement 5 (*p* = .04) were originally low but markedly increased, whereas the increase in the correct rate for statement 2 (*p* = .21) was not statistically significant. Statements 3 and 7 examined the respondents’ understanding of the contents of PC, and the correct rate for statement 7 was notably improved from 49.2 to 70.1% during the clerkship. Statements 8 and 12 examined the respondents’ understanding of the patients’ roles in PC; the correct rates for both before the clerkship were relatively low (30.4 and 66.1%), and the increase in the correct rate for statement 8 (*p* = .00) during the clerkship was statistically significant, while the increase in the correct rate for statement 12 was not significant (66.1 to 70.6%, *p* = .62). Statements 9 and 10 addressed supportive resources required for PC provision, and the correct rate for statement 10 increased significantly (51.8% to71.9, *p* = .04), while the improvement of the correct rate for statement 9 was not statistically significant (*p* = .42).

**Table 2:** Respondents’ understanding of pharmaceutical care

Item	Correct rate (%)		Effect size (%)	*p*-value
	Before clerkship	After clerkship		
[1] PC providers are directly responsible for patients’ clinical outcomes.	482 (80.1)	571 (94.9)	14.8	.13
[2] The primary goal of PC is to maintain and improve patients’ quality of life.	433 (71.9)	512 (85.0)	13.1	.21
[3] The main contents of PC are the provision of drug information. [F]	568 (94.4)	593 (98.5)	4.1	.70
[4] The term “clinical pharmacy” can be replaced with “pharmaceutical care”. [F]	277 (46.0)	413 (68.6)	22.6	.02*
[5] PC is an extension of present community pharmacy services.	410 (68.1)	534 (88.7)	20.6	.04*
[6] In PC, providers identify and deal with patients’ existing and potential drug-treatment problems.	583 (96.8)	595 (98.8)	2.0	.81
[7] PC involves a defined process of activities, in which all steps must be completed to provide this service.	296 (49.2)	422 (70.1)	20.9	.04*
[8] All patients who are treated with drug therapy need PC.	183 (30.4)	357 (59.3)	28.9	.00*
[9] Carrying out PC necessitates drug information support.	464 (77.1)	527 (87.5)	10.4	.42
[10] PC providers need counseling rooms or other private areas to provide PC. [F]	312 (51.8)	433 (71.9)	20.1	.04*
[11] Drug use can be monitored in PC to improve drug treatment.	520 (86.4)	577 (95.9)	9.5	.58
[12] Patients do not need to actively receive PC. [F]	398 (66.1)	425 (70.6)	4.5	.62

^*^ Statistically Significant (*p* < 0.05)

[F] False items

Effect size calculated as the increase of the correct rate of each item after the clerkship

### Respondents’ attitudes toward PC

Table 3 demonstrates that the rates of positive responses to all 14 statements increased after the clerkship. Statements 1, 2, 3 and 4 are connected with the duties of pharmacists in PC. Fewer positive responses for statements 1, 3 and 4 than for the other statements were noted before the clerkship (72.3, 85.2 and 73.3%, respectively); however, the positive response rates for them increased to 95.1, 98.3 and 84.1%, respectively, after the clerkship. For statement 4, the response rate of “disagree” increased after the clerkship. Statements 5, 6, 7, 11, 12 and 13 examined the value and importance of PC. Among these statements, only 6 presented a low positive ratio, of 76.3%, before the clerkship, whereas the other statements exhibited high positive ratios. After the clerkship, all positive responses increased to some degree, especially for statement 6, for which the rates of positive responses increased to 98.9%. Statements 8 and 10 gauge the effects of PC on pharmacy students and pharmacists, and both statements displayed increased positive ratios after the clerkship, from 94.5 to 97.5% and 82.8 to 93.7%, respectively. Statements 9 and 14 explored the acceptance of “working in pharmaceutical care” and “being a clinical pharmacist” by pharmacy students. Before and after the clerkship, the positive rates for these two statements increased from 66.0 and 81.3% to 74.1 and 88.4%, respectively. However, a unique phenomenon observed was that more students responded to statement 9 with “disagree” and “strongly disagree” after the clerkship, with the negative rates was increasing from 10.8 to 14.8%.

**Table 3:** Respondents’ attitudes toward pharmaceutical care

Item	Strongly agree (%)		Agree (%)		Neutral (%)		Disagree (%)		Strongly disagree (%)		*p*-value	Effect size (%)
	Before	After	Before	After	Before	After	Before	After	Before	After		
[1] All pharmacists should perform PC.	172 (28.6)	258 (42.9)	263 (43.7)	314 (52.2)	111 (18.4)	16 (2.7)	48 (8.0)	12 (2.0)	8 (1.3)	2 (0.3)	.03^*^	22.8
[2] The primary responsibility of pharmacists in healthcare settings is to prevent and solve medication-related problems.	241 (40.0)	417 (69.3)	331 (55.0)	171 (28.4)	19 (3.2)	11 (1.8)	9 (1.5)	3 (0.5)	2 (0.3)	0 (0)	.03^*^	2.7
[3] Pharmacists’ primary responsibility is to practice PC.	402 (66.8)	528 (87.7)	111 (18.4)	64 (10.6)	46 (7.6)	5 (0.8)	35 (5.8)	4 (0.7)	8 (1.3)	1 (0.2)	.04^*^	13.1
[4] Undergraduate students majoring in clinical pharmacy are competent to perform PC during their clerkship.	104 (17.3)	358 (59.5)	337 (56.0)	148 (24.6)	139 (23.1)	51 (8.5)	21 (3.5)	33 (5.5)	1 (0.2)	12 (2.0)	.03^*^	10.8
[5] Providing PC is valuable.	497 (82.6)	531 (88.2)	63 (10.5)	66 (11.0)	39 (6.5)	5 (0.8)	2 (0.3)	0 (0)	1 (0.2)	0 (0)	.32	6.1
[6] Providing PC takes too much time and effort. [R]	24 (4.0)	0 (0)	45 (7.5)	3 (0.5)	74 (12.3)	4 (0.7)	172 (28.6)	225 (37.4)	287 (47.7)	370 (61.5)	.03^*^	22.6
[7] I would like to perform PC as a clinical pharmacist.	423 (70.3)	469 (77.9)	155 (25.8)	124 (20.6)	23 (3.8)	9 (2.0)	1 (0.2)	0 (0)	0 (0)	0 (0)	.43	2.4
[8] Providing PC during my clerkship is professionally rewarding.	410 (68.1)	531 (88.2)	159 (26.4)	56 (9.3)	29 (4.8)	12 (2.0)	3 (0.5)	3 (0.5)	1 (0.2)	0 (0)	.08	3
[9] PC is the right direction for my career.	184 (30.6)	305 (50.7)	213 (35.4)	141 (23.4)	140 (23.3)	67 (11.1)	53 (8.8)	82 (13.6)	12 (2.0)	7 (1.2)	.06	8.1
[10] Providing PC would benefit pharmacists.	376 (62.5)	463 (76.9)	122 (20.3)	101 (16.8)	86 (14.3)	30 (5.0)	18 (3.0)	8 (1.3)	0 (0)	0 (0)	.05^*^	10.9
[11] Providing PC will improve patient health.	439 (72.9)	524 (87.0)	140 (23.3)	74 (12.3)	19 (3.2)	3 (0.5)	3 (0.5)	1 (0.2)	1 (0.2)	0 (0)	.15	0.7
[12] Practicing PC will benefit my professional pharmacy career as a clinical pharmacist.	582 (96.7)	597 (99.2)	8 (1.3)	4 (0.7)	12 (2.0)	1 (0.2)	0 (0)	0 (0)	0 (0)	0 (0)	.63	1.9
[13] Providing PC is not worth the additional workload that it places on the pharmacist. [R]	2 (0.3)	0 (0)	1 (0.2)	2 (0.3)	15 (2.5)	12 (2.0)	63 (10.5)	35 (5.8)	521 (86.5)	553 (91.9)	.56	−0.2
[14] I feel proud to be a clinical pharmacist.	355 (59.0)	402 (66.8)	134 (22.3)	130 (21.6)	87 (14.5)	58 (9.6)	26 (4.3)	12 (2.0)	0 (0)	0 (0)	.25	7.1

^*^ Statistically Significant (*p* < 0.05)

[R] Reverse coded items

Effect size is calculated as the increase of the percentage of respondents that strongly agree or agree (strongly disagree and disagree for reverse coded items) each statement after the clerkship

## Discussion

The present study found that the pharmacy students’ understanding of and attitudes toward PC in China were generally improved by the clerkship schemes, but some specific aspects of the clerkship schemes still need improvement, including the training on students’ understanding of the basic conception, the primary goal and the entire processes of PC, and their attitudes toward performing PC as pharmacy students, the necessity of all pharmacists to performing PC and choosing PC provider as their career。.

The results indicated a potential lack of emphasis on shaping students’ basic conceptions of PC in the classroom education, and some of them were not significantly improved by the clerkship, including the PC providers’ directly responsibility for patients’ clinical outcomes [5, 47], the primary goal of PC (maintaining and improving patients’ quality of life [45]), the relationship between clinical pharmacy and PC (PC is an extension and evolution of clinical pharmacy [45]), and the necessary resources for PC provision (private patient counseling area, presence of appropriate drug information references, etc. [7]). It may stem from that hospital pharmacy in China is still in an early stage of transformation from a drug-orientation to patient-orientation [49], for which the drug-oriented responsibilities remains dominating the hospital pharmacy workload and, accordingly, the contents of clerkship schemes, which are usually consistent with the actual hospital pharmacy work, so that the training relevant to PC are insufficient for shaping the conception above. This issue may also be due to the traditional working pattern of hospital medical teams in China that the physicians conventionally dominate the entire process of care from diagnosis to outcome evaluation, while clinical pharmacists are seemingly playing the role of clinical assistants to fulfil the physicians’ clinical needs instead of the patients’, especially when in the clerkship.

The integrated view of PC helps the PC providers identify and specify their roles in the practice setting and their responsibilities in the entire processes of care [44, 47]. Another finding of this study is that the current clerkship schemes did not significantly improve the students’ integrated view, though it is promoted by professional organizations like American Society of Health-System Pharmacist and American College of Clinical Pharmacy, and the Chinese Ministry of Health. The absence of the integrated view of PC may lead to the providers’ neglect of the patients’ or other health care providers’ potential needs. An integrated PC theoretical framework that conforms to the current hierarchical health care system and the pattern of patient care in China [28] and interprofessional education designs in developed countries like the UK [50] could be referential for developing clerkship schemes for pharmacy students in China.

An interesting finding is that some participants’ attitudes toward the opinion “students can perform PC during their clerkship” turned negative after the clerkship scheme, and this phenomenon was possibly caused by two main reasons. On one hand, most clerkship schemes for pharmacy students in China cover the entire processes of hospital pharmacy work, among which the PC is usually more observational than practical to avoid possible errors by the inexperienced students, which may hamper the care or harm the patients, while drug-oriented training items like drug supply, storage management and dispensing are relatively more practical [42]. On the other hand, in most areas of China, drug-oriented responsibilities remain priory to the provision of PC to the hospital pharmaceutical staff, and under the universal shortage of manpower [21], pharmacy students participating in clerkship schemes are usually considered to be a competent “backup” of the pharmaceutical staff. Assigning the pharmacy students to assist in the drug-oriented works meets the needs of practice, but it costs the students’ chances to perform PC and possibly form the wrong understanding of the responsibilities of the clinical pharmacist in their early stage of career. Several studies published in China suggested strengthening the clinical practice of patient information obtaining, medical record reviewing and pharmaceutical information gathering to deepen the students’ participation in PC during the clerkship schemes, but this strategy is yet to be tested [40]. Also, the alternant of classroom education and practice training of Pharm. D programs in the US is a referential design that may improves the students’ self-efficacy of performing PC in the clerkship by enhance the continuity of theoretical and practical knowledge.

This study also found that, although the pharmacy students had generally positive attitudes toward choosing clinical pharmacist as their career, a substantial number of students who previously exhibited positive or neutral attitudes negatively changed their opinion. This paradox might be due to the common status of health care providers in the PC system in China, such as the universal overworking or the relatively low income of pharmaceutical professionals in hospitals, which may markedly reduce the attraction of the clinical pharmacist occupation to the students, and consequentially hamper the faculty development of clinical pharmacists in hospitals. Clarifying career ladders of clinical pharmacy professionals and providing different types of conditional opportunities of further education are feasible strategies for hospitals to retain clinical pharmacy professionals in China [32], and may achieve similar effect on pharmacy students.

This study has some limitations. First, the results showed that the respondents generally had relatively positive attitudes toward PC, and it may due to personal goodwill toward their university and teachers, particularly when certain researchers were not only their teachers but also in charge of their course examinations. Second, China’s present PC system remarkably differs from those of developed countries such as the US and the UK in service pattern, available resources and people’s need of PC, thus our results might not be directly applicable to developed countries.

Future studies can further analyze the specify phenomenon that this study detected, or more in-depth examine the effect of clerkship schemes on improving the pharmacy students understanding of and attitudes toward PC using improved instruments. Further studies can also be enhanced by including pharmacy students from other universities into the sample.

## Conclusion

This study examined the effectiveness of clerkship schemes in China on improving pharmacy students’ understanding of and attitudes toward PC, and found that the current clerkship schemes were generally effective, but some specific aspects of the clerkship scheme still need to be improved for better quality. By attending the clerkship schemes, pharmacy students’ understanding of PC was improved, and they more readily recognized the importance and value of pharmacists providing PC. However, the students’ understanding of some general conceptions of PC and the integrated view of PC were not significantly improved. This study also found that current designs of clerkship schemes may reduce the students’ willingness to perform PC in their early stage of career and reduced the attraction of the clinical pharmacist occupation to the students. Several strategies are referential for improving the current clerkship schemes for pharmacy students in China.

## Supplementary information

**Additional file 1.** English version of original questionnaire.

## Data Availability

The datasets generated and/or analyzed during the current study are not publicly available, because data used in this research is part of a larger data set exclusively constructed with the authors’ efforts, and this data set is to be used in the authors’ other researches, which required confidentiality.

## Abbreviations

CPU: China Pharmaceutical University
PC: Pharmaceutical care
