# Supplementary material for: Do clerkship schemes effectively improve pharmacy students’ understanding of and attitudes regarding pharmaceutical care?——a pre-post study in China Pharmaceutical University
Source: BMC Med Educ. 2019 Nov 15;19:425. doi: 10.1186/s12909-019-1862-x (PMC6858655; doi:10.1186/s12909-019-1862-x)
Supplement: Supplementary file 1 — Additional file 1. English version of original questionnaire. [file 12909_2019_1862_MOESM1_ESM.docx]

Additional file 1 English version of original questionnaire

**Questionnaire for the Clinical Pharmacy Clerkship Schemes**

**National Development Research Center of Licensed Pharmacist**

**China Pharmaceutical University**

Dear students from clinical pharmacy major of China Pharmaceutical University:

Welcome to this survey!

We are researchers from National Development Research Center of Licensed Pharmacist, China Pharmaceutical University. This is a questionnaire survey for evaluating the effectiveness of the clinical pharmacy clerkship schemes on improving the understanding of and attitudes toward pharmaceutical care (PC). The aim of this survey is to provide advices and reference for improving the clerkship schemes for better quality. Please finish the questionnaire according to your actual understanding of and attitudes toward PC.

The information and data obtained in this study are for scientific research only and will not be used for any commercial purpose. Thank you for your support and cooperation!

All questions below are blank filling or single choice.

| ID number | 001 | Registration year | 2014 |
| --- | --- | --- | --- |
| Gender | □male □female □other | Clerkship hospital level | □Secondary □Tertiary |
| Your age |  | Your current GPA |  |
| Do you have internship or work experience involving PC？ | | | □Yes □No |
| If you are filling the questionnaire after clerkship, please provide your clerkship grade | | |  |
| Is each following statement true or false? | | | |
| [1] PC providers are directly responsible for patients’ clinical outcomes. | | | |
| □True □False | | | |
| [2] The primary goal of PC is to maintain and improve patients’ quality of life. | | | |
| □True □False | | | |
| [3] The main contents of PC are the provision of drug information. | | | |
| □True □False | | | |
| [4] The term “clinical pharmacy” can be replaced with “pharmaceutical care”. | | | |
| □True □False | | | |
| [5] PC is an extension of present community pharmacy services. | | | |
| □True □False | | | |
| [6] In PC, providers identify and deal with patients’ existing and potential drug-treatment problems. | | | |
| □True □False | | | |
| [7] PC involves a defined process of activities, in which all steps must be completed to provide this service. | | | |
| □True □False | | | |
| [8] All patients who are treated with drug therapy need PC. | | | |
| □True □False | | | |
| [9] Carrying out PC necessitates drug information support. | | | |
| □True □False | | | |
| [10] PC providers need counseling rooms or other private areas to provide PC. | | | |
| □True □False | | | |
| [11] Drug use can be monitored in PC to improve drug treatment. | | | |
| □True □False | | | |
| [12] Patients do not need to actively receive PC. | | | |
| □True □False | | | |
| What is your attitudes toward each following statements？ | | | |
| [1] All pharmacists should perform PC. | | | |
| □Strongly agree □Agree □Neutral □Disagree □Strongly disagree | | | |
| [2] The primary responsibility of pharmacists in healthcare settings is to prevent and solve medication-related problems. | | | |
| □Strongly agree □Agree □Neutral □Disagree □Strongly disagree | | | |
| [3] Pharmacists’ primary responsibility is to practice PC. | | | |
| □Strongly agree □Agree □Neutral □Disagree □Strongly disagree | | | |
| [4] Undergraduate students majoring in clinical pharmacy are competent to perform PC during their clerkship. | | | |
| □Strongly agree □Agree □Neutral □Disagree □Strongly disagree | | | |
| [5] Providing PC is valuable. | | | |
| □Strongly agree □Agree □Neutral □Disagree □Strongly disagree | | | |
| [6] Providing PC takes too much time and effort. | | | |
| □Strongly agree □Agree □Neutral □Disagree □Strongly disagree | | | |
| [7] I would like to perform PC as a clinical pharmacist. | | | |
| □Strongly agree □Agree □Neutral □Disagree □Strongly disagree | | | |
| [8] Providing PC during my clerkship is professionally rewarding. | | | |
| □Strongly agree □Agree □Neutral □Disagree □Strongly disagree | | | |
| [9] PC is the right direction for my career. | | | |
| □Strongly agree □Agree □Neutral □Disagree □Strongly disagree | | | |
| [10] Providing PC would benefit pharmacists. | | | |
| □Strongly agree □Agree □Neutral □Disagree □Strongly disagree | | | |
| [11] Providing PC will improve patient health. | | | |
| □Strongly agree □Agree □Neutral □Disagree □Strongly disagree | | | |
| [12] Practicing PC will benefit my professional pharmacy career as a clinical pharmacist. | | | |
| □Strongly agree □Agree □Neutral □Disagree □Strongly disagree | | | |
| [13] Providing PC is not worth the additional workload that it places on the pharmacist. | | | |
| □Strongly agree □Agree □Neutral □Disagree □Strongly disagree | | | |
| [14] I feel proud to be a clinical pharmacist. | | | |
| □Strongly agree □Agree □Neutral □Disagree □Strongly disagree | | | |
